# Feasibility of real-time three-dimensional stress echocardiography: pharmacological and semi-supine exercise

**DOI:** 10.1186/1476-7120-8-10

**Published:** 2010-03-24

**Authors:** Lorenza Pratali, Sabrina Molinaro, Anca I Corciu, Emilio M Pasanisi, Marco Scalese, Rosa Sicari

**Affiliations:** 1 CNR, Institute of Clinical Physiology, Pisa, Italy

## Abstract

**Background:**

Real time three dimensional (RT3D) echocardiography is an accurate and reproducible method for assessing left ventricular shape and function.

**Aim:**

assess the feasibility and reproducibility of RT3D stress echocardiography (SE) (exercise and pharmacological) in the evaluation of left ventricular function compared to 2D.

**Methods and results:**

One hundred eleven patients with known or suspected coronary artery disease underwent 2D and RT3DSE. The agreement in WMSI, EDV, ESV measurements was made off-line.

The feasibility of RT-3DSE was 67%. The inter-observer variability for WMSI by RT3D echo was higher during exercise and with suboptimal quality images (good: k = 0.88; bad: k = 0.69); and with high heart rate both for pharmacological (HR < 100 bpm, k = 0.83; HR ≥ 100 bpm, k = 0.49) and exercise SE (HR < 120 bpm, k = 0.88; HR ≥ 120 bpm, k = 0.78). The RT3D reproducibility was high for ESV volumes (0.3 ± 14 ml; CI 95%: -27 to 27 ml; p = n.s.).

**Conclusions:**

RT3DSE is more vulnerable than 2D due to tachycardia, signal quality, patient decubitus and suboptimal resting image quality, making exercise RT3DSE less attractive than pharmacological stress.

## 

Two-dimensional SE is an established and validated method for both diagnosis and prognosis in patients with known or suspected coronary artery disease [1-5]. However, grounds for an accurate interpretation in SE rely on two important features: firstly, acoustic windows that allow complete endocardial border visualization within proper planes of the left ventricle (LV) and secondly, prompt acquisition of peak images pertaining to predictive accuracy. The introduction of RT-3D permits single-window acquisition of complete LV segments in a volume-shaped cine-loop [6], and may have the prerequisite to benefit SE. Furthermore, a high level of operator skill is not required to obtain diagnostic quality RT-3D images and as demonstrated in other studies, the total study time needed to complete an RT-3D SE is shorter than for 2D stress echocardiography [7-12]. The aim of this study was to assess the feasibility and reproducibility of RT-3D SE in the evaluation of regional and global left ventricular function: WMSI, EDV, ESV of left ventricle, compared to conventional 2D echo in a high-volume stress echo laboratory employing all types of stressors: dobutamine, dipyridamole, and semi-supine exercise.

## Methods

### Patient population

The study population consisted of 111 (age 64 ± 11 years, 72 males) unselected consecutive patients evaluated prospectively in the echo lab of the Institute of Clinical Physiology from April 2007 to February 2008, and who underwent stress echocardiography for known or suspected coronary artery disease. Indication for stress echocardiography was suspected coronary artery disease. Inclusion criteria were: age ≥ 18 years; adequate echocardiogram to assess regional wall motion in 2D (the echocardiogram was considered adequate if ≥ 13 of the 17 segments were visualized in at least one projection). The stressor used (51 exercise, 31 dipyridamole and 29 dobutamine) was chosen on the basis of specific contraindications, local facilities and physician's preferences. Pharmacological stress echocardiography was used when patients were unable to exercise maximally, or when the exercise electrocardiography result was not diagnostic or was inconclusive. Stress echocardiography were performed on anti-anginal medical therapy in 66 subjects (60%) (β-blockers in 53, calcium-antagonists in 20, or nitrates in 15) and off therapy in 45 (40%) patients. Informed consent was obtained from all patients before testing, and the study protocol was approved by the institutional review board.

#### 2D imaging

2D echocardiography images were obtained with iE 33 (Philips Medical Systems, Palo Alto, Calif., USA) equipped with a phased array 1.6-2.5 MHz probe with second harmonic capability. In all patients, four standard views (parasternal long and short axis, apical 4 and 2 chambers) were obtained at baseline and peak stress and were digitally stored.

#### RT 3D imaging

Real-time three-dimensional echocardiography images were stored using a matrix-array transducer (X3-1, 1,9/3,8 MHz) connected to a commercial ultrasound machine (IE 33, Philips Medical Systems, Andover, Mass) at baseline and peak stress immediately after 2D acquisition. The RT3D acquisitions were performed in apical view, taking care to optimize the image quality and to include the entire LV cavity within the pyramidal volume scan by using the biplane format. For each patient three different RT3D echo acquisitions were made in standard apical view. RT3D data sets were acquired during breath-holding using a wide-angle acquisition (92 × 83 degrees) mode in which four wedge-shaped sub volumes were obtained from four consecutive cardiac cycles with the acquisition triggered to the R wave of the ECG. Before storage, the visualization of LV endocardial borders in the orthogonal and transverse planes was checked. 3D data sets were transferred to a computer for off-line analysis using available commercial software (3DQ ADV, Qlab version 5.0, Philips). Off-line qualitative and quantitative analyses of regional LV function were performed by two investigators experienced in interpreting RT3D images in standard apical views, and who were unaware of the results of all prior evaluation. Qualitative analysis of 3D LV regional function was done by visual evaluation of wall motion performed on 2D planes reconstructed from the pyramidal volume data set cropped so as to reproduce the 4 and 2 chambers and scored by WMSI. Briefly, from the automatically-traced endocardial borders (manually corrected when indicated) in all frames of the cardiac cycle, a global LV function curve was generated to obtained end-diastolic volume, end-systolic volume, and ejection fraction. The left ventricle was divided into 17 segments from base to apex and regional volume changes were computed over the cardiac cycle and displayed as waveforms. Acquisition of baseline and peak 3D images were obtained after 2D studies using the same echocardiographic machine with a rapid switch between the two probes.

#### Stress protocol

Exercise stress echo was conducted using a semi-supine bicycle ergometer with 25 W incremental loading every 2 min. Dipyridamole (up to 0.84 mg over 6 min) and dobutamine (up to 40 mg/kg/min with co-administration of atropine up to 1 mg) stress echo were performed according to the well-established protocols [13]. Echocardiographic images were semi-quantitatively assessed using a 17-segment, 4-point scale model of the left ventricle [13]. A wall motion score index was derived by dividing the sum of individual segment scores by the number of interpretable segments. Ischemia was defined as stress-induced new and/or worsening of pre-existing wall motion abnormality, or biphasic response (i.e. low-dose improvement followed by high-dose deterioration). Necrotic pattern was akinetic or dyskinetic myocardium with no thickening during stress. A test was normal in the case of no rest and stress wall motion abnormality.

### Image review and analysis

Segmental analysis (wall motion and image quality) for 2D and for RT-3D stress echo was independently and blindly reviewed off-line by two expert echocardiographers. The quality was assessed for each stress test both for 2D and 3D echocardiogram on a three-point scale: 2 optimal/good = complete endocardial definition and wall thickening with exactly similar image views for 2D and 3D echocardiogram; 1 fair = inadequate visualization of one or two segments but adequate visualization of adjacent segments within the same territory; 0 poor = inadequate visualization of three or more segments of the same territory but adequate visualization of adjacent segments within the same territory.

### Statistics

Continuous variables were expressed as the mean value ± SD, and as numbers (percent) for categorical variables. Correlations were performed with linear regression and Pearson's coefficient.

The 2D and 3D measurements were evaluated by calculating the intra- and inter-observer variability of each technique, which was defined as the absolute mean of the difference between the corresponding repeated measurements. The reproducibility between 2D and 3D measurements, 3D observer 1° and 3D observer 2° measurements were evaluated using Bland-Altman analysis by calculating the bias (mean difference) and the 95% limits of agreement (1.96*SD around the mean difference). The significance of the biases was tested through the use of paired Wilcoxon tests with a two-sided alternative. For the analysis of inter-observer RT3D echocardiographic variability, we calculated an adjusted coefficient of variation (CV), defined as the ratio of the SD and the mean of absolute readings for each echocardiographic parameter. Agreements between results obtained by the 2D and 3D approaches were tested using Cohen's coefficient of variation, Kappa statistics, and with segment-to-segment comparison. A kappa statistic less than 0.40 was considered to represent poor agreement, between 0.40 and 0.75 fair to good agreement, and greater than 0.75 excellent agreement. The 95% CIs were calculated for each technique, and the individual intervals were compared. Differences between techniques were considered significant at the 0.05 level when 95% CI did not overlap. All calculations were made using SPSS software (SPSS version 13.0 for Windows, 2004).

## Results

### Feasibility of imaging and population characteristics

**Figure 1:**
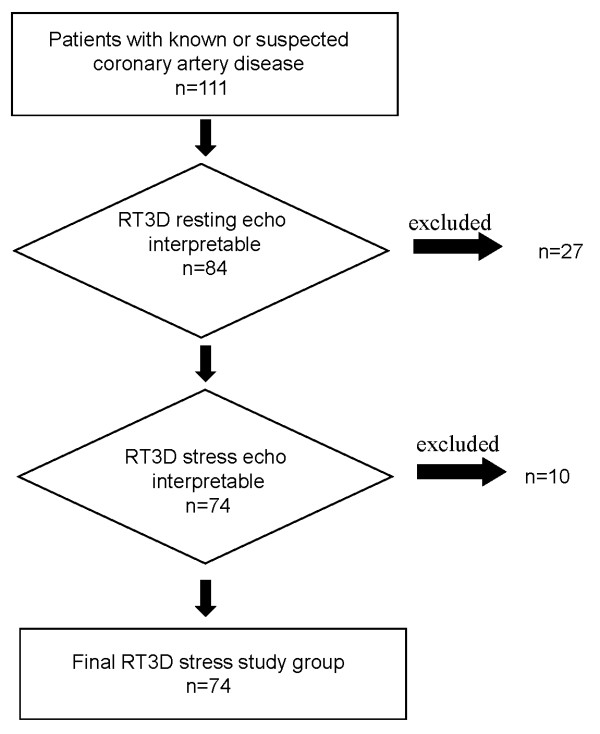
**RT3D feasibility at rest: 27 patients were excluded from analysis because of translational artefact or for un-interpretable images**. Ten patients were excluded for un-interpretable images at peak stress. Of 37 RT3D echo tests excluded from analysis, 25 were exercise and 12 pharmacological tests.

One hundred eleven consecutive patients underwent 2D and RT3D stress echocardiography (51 exercise echo, 31 dipyridamole echo, and 29 dobutamine echo) for known or suspected coronary artery disease and or viability identification. Ten patients (10%) were excluded for suboptimal acquisition of 3D images for translational artifact or for atrial or ventricular arrhythmia precluding analysis of left ventricular volume. RT3D images were sub-optimal at rest in 17 patients (15%) (additional file 1, 2 and 3) and at peak stress in 10 patients (9%) for other reasons such as patient decubitus during acquisition and/or artifacts at peak stress, high heart rate to preclude endocardial border delineation for accurate assessment of left ventricular volume and WMSI (additional file 4). These 37 patients were excluded from the analysis. Therefore, the overall feasibility of RT3D stress echo was 67%, higher for pharmacological stress echo (12 excluded subjects/60 subjects) than for exercise (25 excluded subjects/51 subjects): 80% vs. 51% p < .0001 (figure 1). Clinical and echocardiographic characteristics of the 74 patients with interpretable 2D and RT3D images constituting the study population are shown in Table 1.

**Table 1:** Clinical and RT3D echocardiographic patient characteristics

Variables	n = 74 pts
age (years)	64 ± 11
male	47 (63%)
hypertension	44 (59%)
diabetes	16 (21%)
hyperlipemia	43 (58%)
smoke	38 (51%)
	
**Coronary disease**	
MI	32 (43%)
PCI	25 (33%)
CABG	7 (9%)
Angiographic results: 31 pts	
normal vessels	18
1-vessel disease	5
2-vessel disease	3
3-vessel disease	5
	
**Medical therapy**	
ACE inhibitors	46 (62%)
Beta-blockers	41 (55%)
Nitrates	18 (24%)
Calcium channel blockers	16 (21%)
Aspirin	51 (69%)
Statins	36 (48%)
	
**RT3D echo**	
Rest LVED volume (ml)	119 ± 59
Rest LVES volume (ml)	64 ± 48
Peak LVED volume (ml)	111 ± 54
Peak LVES volume (ml)	58 ± 42

MI: myocardial infarction, PCI; Percutaneous coronary interventions;

CABG; Coronary artery bypass graft; LVED: Left ventricular end-diastolic;

LVES: Left ventricular end-systolic.

### RT3D stress test echo

**Figure 2:**
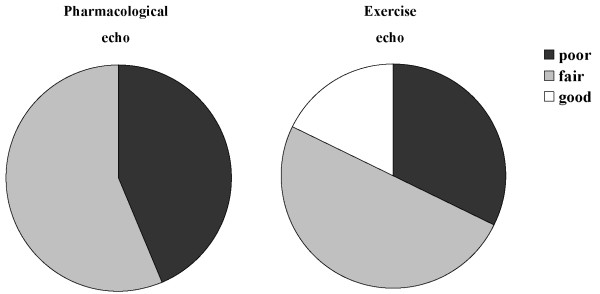
**Distribution of patients according to RT3D image quality separating the study group into pharmacological and exercise stress echo**.

No complications occurred during stress echocardiography. Out of 74 patients, 28 subjects (38%) underwent semi-supine exercise, 23 subjects (31%) dipyridamole test, and 23 subjects (31%) dobutamine test. The mean frame rate of RT3D echo during acquisition was 19 ± 3.8 Hz at rest and 18 ± 2.7 Hz at peak stress (p = n.s.). The increase in heart rate during the three different stressors is reported in Table 2. The mean WMSI at rest was 1.31 ± 0.4 by 2D echo and 1.33 ± 0.43 by RT3D echo (mean difference 0.01, 95% CI: -0.41 to 0.38, p = 0.2). At peak stress the mean WMSI was 1.26 ± 0.4 by 2D echo and 1.25 ± 0.4 by RT3D echo (mean difference 0.01, 95% CI: -0.39 to 0.41, p = 0.6). The two methods showed an excellent agreement in WMSI at rest (Pearson's correlation index 0.957, CI 95%: 0.85 to 0.98; p < .0001) and at peak stress (Pearson's correlation index 0.942, CI 95%: 0.85 to 1; p < .0001). The RT3D image quality was deemed to be good/fair in 44 patients, and only poor but adequate in 30 patients, and it was better in patients submitted to exercise echo in comparison to pharmacological stress (figure 2). This difference is due to the fact that in our clinical practice, patients with sub-optimal acoustic window are shifted to pharmacological stress echocardiography.

**Table 2:** Heart rate at rest and peak of stress

Test	Heart rate: Rest (bpm)	Heart rate: Peak (bpm)
**Exercise**	73 ± 15	127 ± 23
**Dipyridamole**	67 ± 9	80 ± 10
**Dobutamine**	68 ± 13	104 ± 20

### 2D and RT3D analysis and reproducibility of wall motion of left ventricle

With 2D echocardiography, of all the potential 1241 segments, 1214 (97.8%) were visualized at rest and 1146 (92.3%) at peak stress. The inter-observer agreement for 2D echo was good at rest (kappa value = 0.75) and at peak stress (kappa value = 0.70).

For 3D echo 1206 (97%) segments were visualized at rest and 1108 (89%) at peak stress and the agreement between the two different observers for 3D echo was excellent at rest (kappa value 0.78) and at peak stress (kappa value = 0.75).

**Figure 3:**
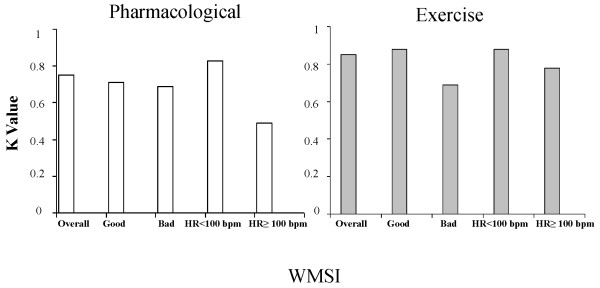
**Agreement between two different observers for the evaluation of RT3D WMSI considering two different groups: exercise and pharmacological stress echo**. In the graph the K value according to image quality and heart rate at peak stress is reported.

We evaluated the agreement between the two observers by separating the total population according to image quality at peak stress and patient decubitus during the stress (semi-supine decubitus for exercise and left lateral decubitus for pharmacological stressors). In the presence of a bad quality image the agreement between the two observers decreases significantly in the case of semi-supine decubitus (k value = 0.88 with good quality images and 0.69 with bad quality images). Moreover, when patients reached high heart rates, agreement between the two observers significantly decreased both in left lateral and semi-supine decubitus (k value: pharmacological stress HR < 100 bpm = 0.83; HR ≥ 100 bpm = 0.49; Exercise stress HR < 100 bpm = 0.88; HR ≥ 100 bpm = 0.78) (figure 3).

### LVEDV, LVES by RT3D echo

**Figure 4:**
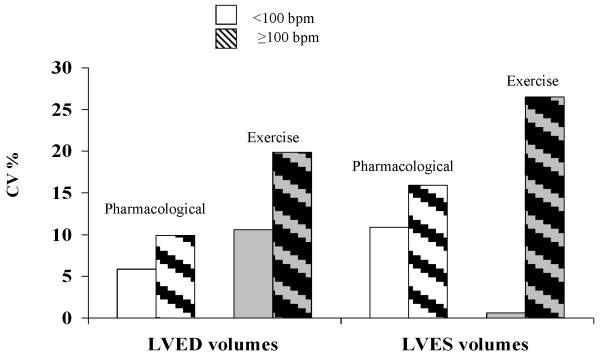
**Coefficient of variation for LV ED and ES volumes in pharmacological and exercise stress at different heart rate values at peak stress**.

RT3D measurements of LV volumes were feasible in all patients (Table 1). In the population study there was an excellent inter-observer agreement with the Bland Altman method with mean ± SD for LVEDV at rest (2.3 ± 18 ml; CI 95%: -38 ml to 34 ml; p = n.s.) and at peak stress (5.8 ± 16 ml; CI 95%: -38 ml to 26 ml, p = 0.002), LVESV at rest (3.6 ± 23 ml; CI 95%: -48 to 41 ml; p = 0.03) and at peak stress (0.3 ± 14 ml; CI 95%: -27 to 27 ml; p = n.s.). Moreover, we evaluated the inter-observer variability considering two different groups: group A submitted to stress echo in left lateral decubitus (pharmacological stress) and group B: semi-supine decubitus (exercise stress); these two groups were further separated on the basis of heart rate at peak stress. Variability analyses of RT3D echocardiographic parameters are shown in Table 3 and figure 4. The variability was high for the LVEDV during exercise echo at high heart rates (CV: 20%) and for LVESV, in both pharmacological and exercise echo, especially with high heart rate (CV: 15.9% for pharmacological echo and 26.5% for exercise echo). The variability was low for LVED and LVES volumes both for pharmacological and exercise echo at low heart rates at peak stress.

**Table 3:** Inter-observer variability summary

	Inter-observer CV%	
**RT3D echo measure**	***Pharmacological echo***	***Exercise echo***
Rest LVED volume (ml)	6.1	11.2
Peak LVED volume (ml)	0.1	12.5
Rest LVES volume (ml)	4.6	17.2
Peak LVES volume (ml)	0.2	3.9

LVED: Left ventricular end-diastolic; LVES: Left ventricular end-systolic.

In a subset of study patients (n = 17) the intra-observer variability was calculated. The 3D analyses of observer 1 were compared with those previously calculated in a time-lag ranging from 12 to 18 months by the same observer. In this subgroup the agreement was excellent with the Bland Altman method with a mean ± SD for LVEDV at rest (4.7 ± 11 ml; CI 95%: -27 ml to 17 ml) and at peak stress (5 ± 15 ml; CI 95%: -34 ml to 24 ml), LVESV at rest (3.4 ± 7 ml; CI 95%: -17 to 10 ml) and at peak stress (1.17 ± 7.69 ml; CI 95%: -16.2 to 13.9 ml).

## Discussion

RT3D SE is a feasible technique when applied to consecutive patients evaluated for known or suspected coronary artery disease, but some limitations of its performance have to be acknowledged. The resolution of the images was not as high as that obtained with conventional scanners, due to the lower frame rates. The American Society of Echocardiography recommends that 20 frames/s are needed in the majority of examinations, for digital capture and playback at normal heart rate, and that frame rate should be increased to 30 frames/s when heart rate is > 140 bpm, [14]. Real Time 3D scans are at a speed of 18 to 40 frames/s as determined by depth settings. The frame rate at 15 cm depth setting is 20 frames/s. Thus the low frame rate may limit the feasibility of RT3D SE. In our study the mean frame rate was low at rest and at peak stress and this may account for the low feasibility of the RT3D SE, which is only 67%. The position of the patient (semi-supine worse than lateral decubitus) during acquisition could also contribute to the low feasibility; as a matter of fact all the patients excluded from analysis underwent exercise stress echo which is performed in semi-supine decubitus. An other important limitation for feasibility and reproducibility in RT3D SE was the high heart rates reached at peak stress. As demonstrated in the present study, the agreement of WMSI assessment between two independent observers decreased significantly when heart rate was independent of patient decubitus during stress test. In this study we also evaluated the feasibility and reproducibility of LVED and ES volumes at rest and at peak stress when different stressors are used. The LV volume measurements by 3D have a low or moderate inter-observer and intra-observer variability at low heart rates at peak stress. In conclusion during RT3D SE, wall motion analysis is blunted by unacceptably low reproducibility when exercise is the stressor of choice, with resting images of only adequate quality and _ even for resting images of good quality _ when heart rate exceeds 100 b/m. In this study the quantification of left ventricular volumes were reproducible during stress also when heart rate was high or resting images of sub-optimal quality. However, when applied on a consecutive patients population, the overall diagnostic accuracy of RT3D stress echocardiography is significantly lower than 2D.

### Comparison with preview studies

Zwas et al [12] had demonstrated that with an earlier generation of 3D scanners, treadmill stress testing is feasible in healthy volunteers. With the first-generation 3D echocardiography two studies showed that RT3D dobutamine SE showed similar sensitivity as 2D dobutamine echo for the detection of myocardial ischemia [7,11]. Matsumura et al. [8] using a second-generation RT3D echo system showed a trend toward a better sensitivity for RT3D echo than 2D echo in the left anterior descending artery territory, using single-photon emission computed tomography as the reference standard. Also in the study published by Aggeli C et al [15], RT3D echo identified wall motion abnormalities more readily in the apical region than 2D echo and in this study the results were validated with coronary angiography. Only one study has shown that RT3D dipyridamole stress echocardiography is highly feasible with a high concordance rate with 2D standard stress echo [16]. Consistently with previous studies, in this paper [16] it was shown that 2D images need a longer time of acquisition but that RT-3D is more time-consuming for analysis.

**Clinical implications **RT3D has entered the clinical arena but no additional advantage over conventional 2D echocardiography during SE can be demonstrated. The recent EAE [13] consensus statement on SE did not recommend the routine use of this technology, even though it may significantly shorten time of acquisition counterbalanced by a longer time of data-set analysis. Clinically driven application of RT3D should be based mainly _ if not only_ on robust indices incorporating LV volumes (such as ventricular and vascular elastance or mean diastolic filling rates). 2D echo outperforms RT3D echo for regional wall motion assessment but the latter is better for global indices of left ventricular function. Several conclusions can be drawn on the basis of the present results: first, not all patients were created equal: in case of sub-optimal image quality at rest, feasibility is lower for exercise and patients should be re-directed to pharmacological tests. Second, not all stressors were created equal: at peak stress, exercise is less feasible than pharmacological stressors, mostly due to tachycardia image degradation. Lastly, during stress, not all parameters were created equal: the worst reproducibility was obtained for wall motion analysis, the best for LV ESV and EDV at low heart rate (HR < 100 bpm).

## Abbreviations

RT3D: real time three-dimensional; SE: stress echocardiography; WMSI: wall motion score index; EDV: end-diastolic volume; ESV: end-systolic volume; HR: heart rate; 2D: two-dimensional; LV: left ventricle; EAE: European association of echocardiography.

## Competing interests

The authors declare that they have no competing interests.

## Authors' contributions

L.P. designed the study, acquired images, analysed data, and drafted the manuscript; SM and MS analyzed and interpreted data; A.I.C., E.M.P. acquired images and contributed to data collection; RS revised the manuscript critically for important intellectual content. All Authors have read and approved the final manuscript.

## Supplementary Material

Additional file 1 **example movies of good quality movie at rest with RT3D echo**. good quality image of a 4-chamber left ventricle. Click here for file

Additional file 2 **example movies of suboptimal quality movie at rest with RT3D echo**. sub-optimal image due of a 4-chamber left ventricle to artefacts. Click here for file

Additional file 3 **example movies of uniterpretable movie at rest with RT3D echo**. uninterpretable image of a 4-chamber left ventricle for bad acoustic window. Click here for file

Additional file 4 **example movies of good acoustic window at rest both 2D and 3d acquisitions, and uninterpretable echo images at peak stress**. Patient with optimal acoustic window in rest condition (left part of movie) but uninterpretable images at peak stress (right part of movie). Upper part 2D acquisition, lower part 3D acquisition. Click here for file
